# Viabahn stent graft in the management of a grade 3 coronary perforation

**DOI:** 10.1186/s42155-019-0050-8

**Published:** 2019-01-17

**Authors:** Conrad von Stempel, Hossam Fayed, John Antony Goode, Sundeep Kalra, Niket Patel

**Affiliations:** Royal Free London NHS Foundation Trust, London, UK

**Keywords:** Coronary artery perforation, Stent graft, Multi disciplinary team working

## Abstract

**Background:**

Coronary artery perforation during coronary intervention has high morbidity and mortality. This case describes the collaboration between interventional cardiologists and Interventional radiologists to successfully deploy a peripheral arterial stent graft in a coronary artery that demonstrated persistent extravasation after coronary specific stent graft placement.

**Case presentation:**

An 84 year old female patient presented with acute coronary syndrome and coronary angiography identified a right coronary artery lesion. This was dilated and stented but resulted in a grade 3 coronary perforation. Conservative treatment with balloon tamponade failed, as did placement of a covered coronary-specific stent graft. A Viabahn peripheral arterial stent graft was placed within the indwelling stents and successfully sealed the endoleak. At 6 months the patient is clinically well and follow-up imaging has demonstrated stent patency.

**Conclusions:**

In the emergency setting when coronary artery perforation fails to respond to standard initial and bail out techniques, peripheral arterial techniques and devices can be extremely useful. A good relationship between interventional cardiology and radiology is paramount.

## Background

Coronary artery perforation (CP) is a rare complication of percutaneous coronary intervention (PCI). It has severe consequences including cardiac tamponade, myocardial infarction (MI) and death. This case report describes management of a right coronary artery (RCA) Ellis grade 3 perforation that occurred during percutaneous coronary intervention and did not respond to standard balloon tamponade and coronary specific synthetic covered stent graft placement. A peripheral vascular Viabahn stent-graft was inserted with the help of interventional radiology (IR) team, which successfully sealed the perforation. A single case report describes use of a similar device in a chronic coronary pseudoaneurysm with no documented cases of such a device used in the emergency coronary perforation setting.

## Case presentation

**Fig. 1:**
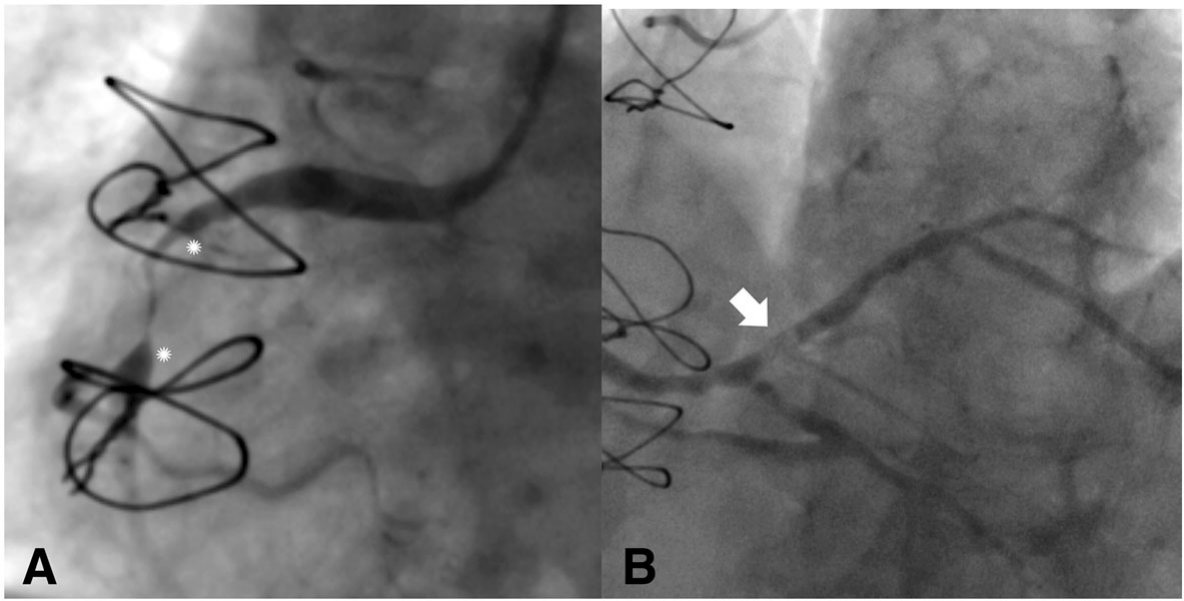
AP projection of RCA angiogram. **a**: Tight stenosis is seen in teh mid-proximal RCA (between asterisks). **b**: Culprit lesion in PLA is a partial filling defect as indicatio by the white arrow

An extremely fit 84 year-old lady attended with acute coronary syndrome with a 6 h history of chest pain. She had a history of hyperlipidemia, hypertension with prior coronary artery bypass in 2004 with a left internal mammary artery graft to the left anterior descending artery and saphenous vein grafts (SVG) to the posterior descending and obtuse marginal arteries. ECG demonstrated inferior ST elevation MI. Preprocedural platelet inhibition with oral Ticagrelor 180 mg and Aspirin 300 mg was administered. Coronary angiography performed from a 6F left radial sheath confirmed severe proximal left coronary disease and patent grafts. The RCA was ectatic with subtotal occlusion of the proximal-mid RCA (see Fig. 1a asterisks) and a further lesion at the ostium of the posterior descending artery (PDA). The culprit lesion was an acute thrombotic lesion affecting the origin of the right posterior lateral artery (PLA) (see Fig. 1b white arrow).

Primary PCI was performed to the thrombotic PLA lesion with pre-dilatation and drug eluting stent (DES, 4.0 × 34 mm Onyx stent). The proximal-mid RCA lesion was predilatated and stented with a 5.0 × 34 mm Onyx.

**Fig. 2:**
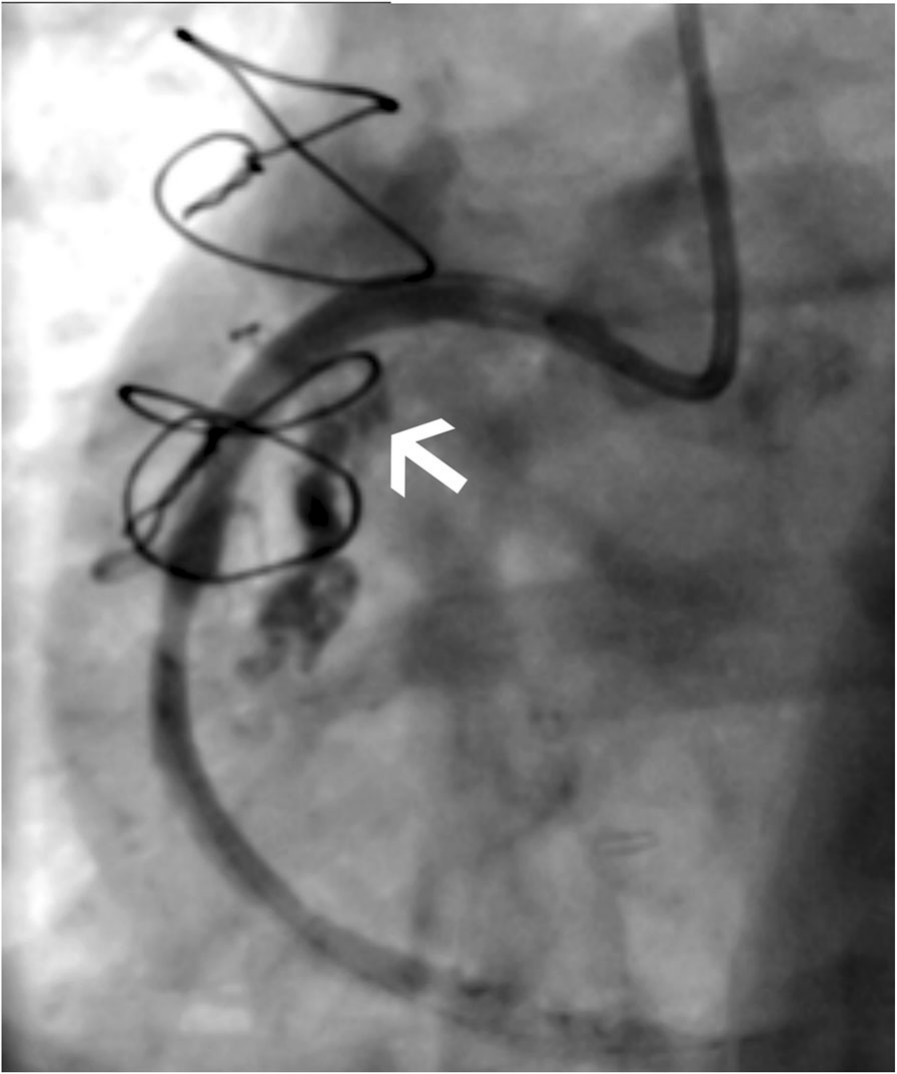
AP projection showing large extravasation of contrast (white arrow)

Following stent deployment the RCA suffered a grade 3 perforation (> 1 mm perforation with streaming of contrast) (Ellis et al., 1994) within the stented segment. Initial management with intermittent prolonged balloon tamponade over 30 min failed. Dual access from the contralateral radial artery was gained to allow ‘ping-pong’ guiding catheters to facilitate rapid exchange of wires and devices minimising tamponade balloon deflation times. The largest coronary covered stent (4.0 × 18 mm Bentley BeGraft) was deployed within the initial DES and over-sized to 4.5 mm by post-dilation with a non-compliant balloon. There was persistent rapid extravasation secondary to an endoleak. Echocardiography confirmed a < 1 cm rim of pericardial fluid and the patient remained haemodynamically uncompromised therefore pericardiocentesis was not indicated. Only limited distal coronary ischemia was seen in view of a patent SVG to PDA supplying the PDA/PLA system via the distal RCA-PLA stent (see Fig. 2).

Given the ectatic nature of the RCA and failed second line techniques, an emergency multi-disciplinary team meeting (MDT) supported from IR colleagues was arranged to consider management. Given the patent right SVG, coil embolization of the RCA was considered. Placement of a peripheral vascular stent-graft was favoured as embolization may have not provided immediate stasis and would leave limited back-up options. An 0.018 in. 300 cm guidewire (Advantage, Terumo) was placed through the stents and an 8Fr glidecatheter from the right radial artery was exchanged. A Viabahn 5 mm × 50 mm stent graft was then deployed within the RCA, through the lumen of the DES and covered stent after rapid removal of the tamponade balloon and wire from the second guiding catheter. The Viabahn was deployed with a proximal and distal flare. Completion angiograms demonstrated successful resolution with preservation of the acute marginal branch and forward flow to the PDA/PLA.

**Fig. 3:**
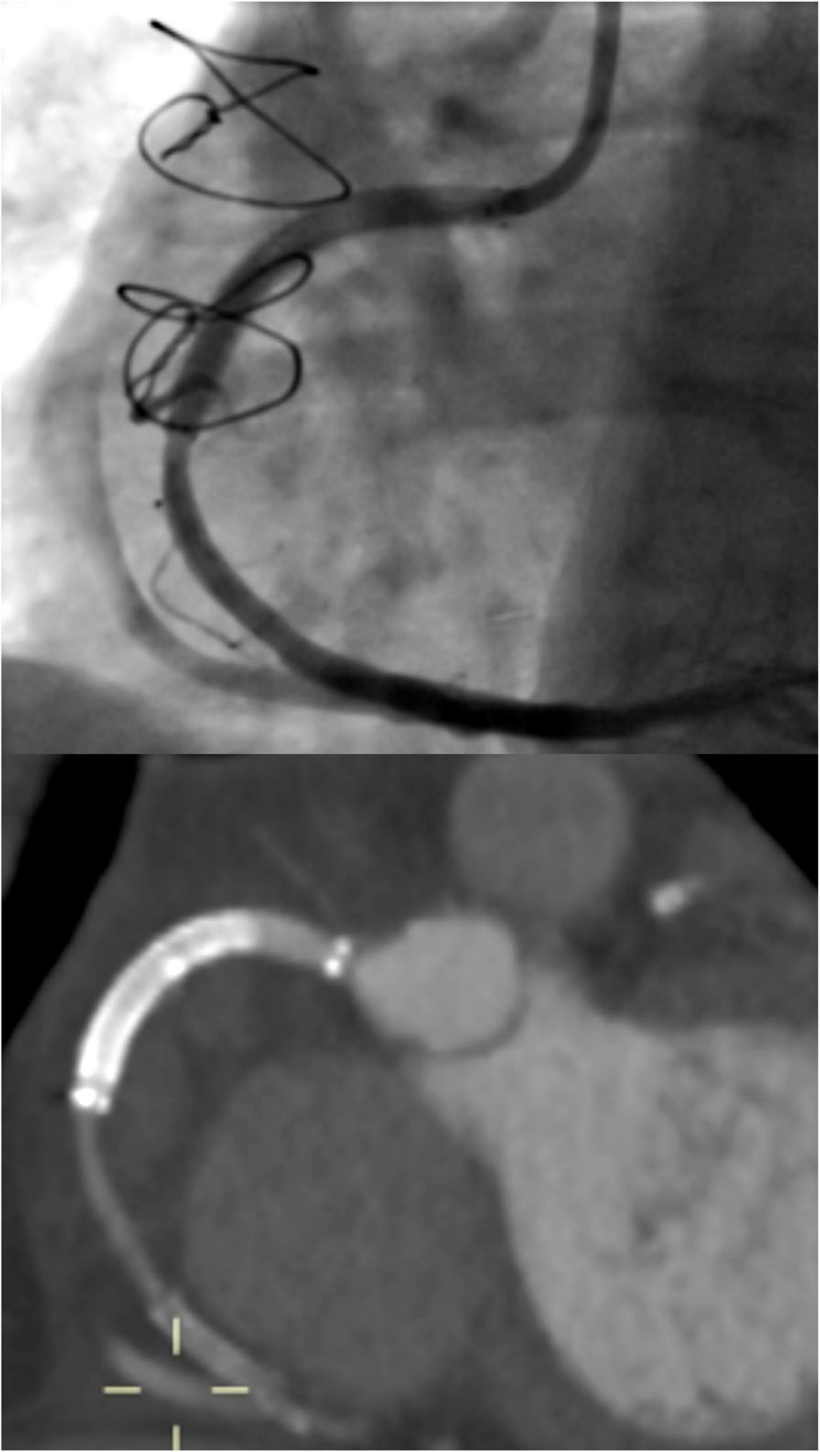
AP projection and CT coronary angiogram showing the Viabahn stent graft in situ with distal patency (reflux of contrast seen into the right SVG)

Twenty-four hours later repeat coronary angiogram was performed which confirmed stent patency, and a CT coronary angiogram at 1 month also shows excellent distal flow in the RCA and it’s branches (see Fig. 3). The patient is asymptomatic at clinical review up to 6 months post-procedure. Repeat echocardiograms did not demonstrate any ventricular aneurysm.

## Discussion

The reported incidence of CP ranges from 0.1% to 3.0% (Ellis et al., 1994; Gunning et al., 2002; Fukutomi et al., 2002; Shimony et al., 2011). It is seen more commonly in Type C stenoses and in older patients with previous coronary intervention or surgical procedures. The RCA is the most common site of perforation (Gunning et al., 2002; Shimony et al., 2011). Perforation can occur secondary to guidewire placement, balloon angioplasty and stent insertion. Stent related CP compromises 21–50% of published series (Shimony et al., 2011; Guttmann et al., 2017). In this case the presumed mechanism of CP was angioplasty after initial DES deployment at the level of a small RV branch arteriole. There was no evidence of strut fracture and the balloon was appropriately sized to the stent.

The classification of perforation proposed by Ellis in 1994 is the most accepted internationally and divides perforations into Grade 1, 2 and 3. Grade 3 CP describes an extravasation jet through a defect of 1 mm in size and free spilling of contrast. These injuries carry up to 20% mortality rate and 10 fold increase in catastrophic tamponade compared with grade 2 (Shimony et al., 2011; Guttmann et al., 2017). The management for CP includes intermittent balloon inflation for 5–15 min, often using dual access to aid rapid interchange of balloons and catheters (Röther et al., 2015). Failing this placement of a stent graft is required or emergency surgery (Javaid et al., 2006).

Stent grafts successfully treat up to 80% grade 3 rupture, but multiple overlapping stents may be required (Lansky et al., 2006; Ly et al., 2005). In this particular case, the ectatic nature of the RCA measuring upwards of 5.5 mm at the ostium and distally 3 mm, meant the largest standard coronary stent-graft failed despite attempted balloon-moulding.

Viabahn stent grafts are covered self-expanding nitinol stents used in peripheral vessels, owing to their flexibility, durability and patency rates. These devices are catheter mounted and usually advanced into the desired position within an introducer sheath, and then revealed once in position. Once revealed, the device should not be re-withdrawn into the sheath. The aim of the introducer sheath is to avoid the device becoming snagged on calcified atheroma as manipulation can result in the device deploying prematurely of causing separation of the mounting catheter. Device delivery is achieved by pulling a drawstring at the device sheath hub. In this case, the RCA already had 2 overlapping stents. As a result the inner lumen was too constrained to allow a parent sheath to pass through; given the emergency nature of this case the Viabahn device was gently advanced into position through the preexisting stents not inside the a parent sheath. This was achieved with slow advancement of the device and magnified high-pulse rate fluoroscopy. Advantage wire provided a sufficiently stable platform to safely perform this maneuver. In the reviewed literature there is a single other case of a 5 mm Viabahn stent used to treat a coronary pseudoaneurysm. The authors describe the flexible self-expanding nature of the stent provides good apposition within the vessel (Kim et al., 2017). In this case the Viabahn stent flared at either end, sealing the endoleak.

## Conclusions

This case demonstrates the importance of robust management algorithms in CP. Despite the failure initial treatments, the rapid response as an MDT and use of dual access sheaths meant bleeding was minimal. Furthermore an excellent rapport with IR colleagues facilitated an emergency multidisciplinary discussion within the angiosuite and the merits of different techniques and devices including peripheral arterial stents and guidewires explored.

## Abbreviations

CP: Coronary Artery Perforation
IR: Interventional radiology
MDT: Multi-disciplinary team meeting
MI: Myocardial infarction
PCI: Percutaneous coronary intervention
PDA: Posterior descending artery
PLA: Posterior lateral artery
RCA: Right coronary artery
SVG: Saphenous vein graft
